# Just do the ECV, Just do it - This, I call it a Miracle:” Understanding External Cephalic Version Practices at the Largest Teaching Hospital in Ghana

**DOI:** 10.21203/rs.3.rs-8347543/v1

**Published:** 2025-12-30

**Authors:** Dhanalakshmi Thiyagarajan, Yana Astter-Sompel, Thelma Quarshie, Jessica McCoy, Mackenzie Woock, Cheryl A. Moyer, Alim Swarray-Deen, Samuel A. Oppong, Emma R. Lawrence

**Affiliations:** Michigan Medicine; University of Michigan-Ann Arbor; Korle Bu Teaching Hospital; University of Michigan-Ann Arbor; University of Michigan-Ann Arbor; Michigan Medicine; University of Ghana; University of Ghana; Michigan Medicine

**Keywords:** Avoidable cesarean deliveries, health equity, ECV, obstetrics procedures, obstetrics complications, malpresentation

## Abstract

**Background:**

External cephalic version (ECV) is a proven, low-risk, low-resource, and cost-effective procedure, when performed by a skilled healthcare professional, to reduce cesarean delivery (CD) rates for malpresented fetuses. Information regarding ECV practices in low- and middle-income countries is limited. We sought to understand and evaluate malpresentation counseling, decision-making, and ECV practices from the perspective of clinicians and patients at the largest referral teaching hospital in Ghana.

**Methods:**

We conducted a prospective, multi-modal cohort study of obstetrics patients who were eligible for ECV at Korle Bu Teaching Hospital in Accra, Ghana between July 1 and December 31, 2024. We enrolled pregnant patients who were ≥ 36 weeks gestation diagnosed with malpresentation. After enrollment, the research team directly observed the patients’ antenatal care visits to collect data on the counseling related to their malpresentation diagnosis, ECV if applicable, and obtained delivery information postpartum via their medical record. Postpartum, patients and their clinicians were recruited to complete semi-structured interviews to understand their perspectives and decision-making regarding ECV. We performed descriptive and inferential statistics including cross-tabulation with Chi-square analysis or Fisher’s exact test for categorical variables and independent samples t-test or analysis of variance for continuous variables. Statistical significance was defined at p < 0.05. Interviews were audio-recorded, translated, transcribed, coded, and thematically analyzed.

**Results:**

Among 46 enrolled patients at 36 weeks’ gestation who were eligible for ECV, only 21 (45.7%) were offered ECV, and 17 (37.0%) underwent ECV. History of one previous CD and training and resource limitations were barriers to clinicians offering ECV. Patients refrained from pursuing ECV if they lacked understanding of the procedure and its complications. Overall, patients and clinicians positively perceived ECV and valued its ability to decrease CD rates.

**Conclusions:**

This study demonstrates important opportunities to improve counseling, utilize evidence-based indications to offer ECV to patients with pregnancies complicated by malpresentation, and ensure clinicians—and potentially midwives—are trained to perform ECV. Addressing these opportunities successfully could improve the number of healthcare professionals who practice safe and effective ECV in sub-Saharan Africa and potentially decrease avoidable rates of CD and their associated morbidity and mortality.

## Background

Evidence shows that cesarean delivery (CD) rates above 19% do not decrease maternal morbidity and mortality.^1^ When not medically necessary, CD pose a greater risk to maternal and neonatal health compared to vaginal delivery, especially in low- and middle-income countries (LMICs), where perioperative resources may be limited.^1–3^ In 2020, over 95% of maternal deaths occurred in LMICs, with the majority being preventable. This highlights the urgent need to reassess the current use of CDs in LMICs in reducing maternal morbidity and mortality.^4^ In 2022, the national CD rate in Ghana was 21%,^5^ while 2016 data indicate the CD rate at Korle Bu Teaching Hospital (KBTH), a tertiary referral hospital where the current research was conducted, was 46.9%.^6^

The third most common indication for primary CD is malpresentation, when the fetus is not in a head down position, which affects approximately 4% of term pregnancies.^1,2^ The American College of Obstetricians and Gynecologists and the Royal College of Obstetricians and Gynecologists recommend that external cephalic version (ECV) be attempted for cases of malpresentation to avoid CD and enable a trial of vaginal delivery.^2,3^ ECV is a procedure in which a skilled clinician applies external pressure to the abdomen of a pregnant person with a malpresented fetus to manually rotate the fetus to a cephalic, head-down presentation that facilitates vaginal delivery.^7^

Existing research helps shed light on patient attitudes toward ECV. In one study, patients in the UK report viewing successful ECV as a means to a “natural” birth, framing vaginal delivery as an “achievement” with faster recovery and CD as a last resort for management, specifically for first-time labor.^10^ However, other patients view ECV as an unnatural manipulation of their fetus and have significant concerns about pain levels during the procedure.^10^ One study from Australia reported patient barriers to ECV to include receiving very little or no information from a clinician on the procedure, concerns about fetal safety, and the fact that a vaginal delivery was not guaranteed after successful ECV.^11^ In another study, some patients reported learning more about ECV from family, friends, and the print media than from a clinician, indicating a possible gap in quality information or an overestimation of risks.^12^ They also found that patients generally responded favorably to “de-mything” negative assumptions about ECV, noting the importance of timing, manner, and depth of information given during malpresentation counseling as important predictors of women’s anxieties surrounding ECVs.^12^ From a clinician’s perspective, several barriers to providing quality ECV counseling exist, including lacking confidence in the skills of the clinician performing the ECV, disagreement among clinicians on contraindications, and a perceived inability to convince patients who have previously decided on planned CD to consider alternative options, as identified by research in the Netherlands.^13^

While these studies shed light on malpresentation counseling, decision-making, and ECV practices in high-income countries, there is very little current information regarding ECV practices and decision-making among patients and clinicians in LMICs. While studies suggest an overwhelming desire for vaginal delivery over CD among women in LMICs,^14^ data from 2009 shows that only 21–52% of healthcare professionals in sub-Saharan Africa perform ECVs.^9^

The aim of the current study was to evaluate the current state of malpresentation counseling and decision-making and ECV practices in Ghana from the perspective of clinicians and patients. Optimizing management of malpresentation and by extension, ECV, into common practice in sub-Saharan Africa has the potential to reduce avoidable CD and its associated morbidity and mortality.

## Methods

We conducted a multi-modal study at the Korle Bu Teaching Hospital (KBTH), in Accra, Ghana. The quantitative component was a prospective, cohort study of obstetrics patients who were eligible for ECV between July 1 and December 31, 2024. In the qualitative component of the study, we conducted semi-structured interviews of clinicians and postpartum patients. KBTH, located in the capital city of Accra, is the largest referral teaching hospital in Ghana and the third largest in sub-Saharan Africa. The 6-floor maternity unit has 275 inpatient beds and performs approximately 8,000 deliveries annually. While updated data is limited, 9% of CDs at KBTH in 2010–2011 were due to malpresentation.^4^ Patients were included if they were receiving antenatal care at KBTH; able to speak English, Twi, or Ga (the two most popular local dialects in the area); and diagnosed with malpresentation at 36 weeks’ gestation or greater. Patients were excluded if they were younger than age 18, referred from another facility for delivery, or had an absolute clinical contraindication to trial of vaginal delivery (which by hospital policy includes a history of two previous CDs).

Institutional review board approval was obtained from KBTH (KBTH-STC/IRB/00044/2024). The study aims were presented to the Department of Obstetrics and Gynecology during their department meeting. Recruitment was completed by trained research assistants at two key hospital locations: the Fetal Assessment Center, where all obstetric ultrasounds are performed, and the outpatient department, where antepartum patients receive outpatient care. Clinicians and nurses attending to these patients notified the research team of potential participants. The research team assessed eligibility by reviewing the patient’s pregnancy care booklet, which is the medical record completed by the patient’s clinician team. If eligible, the study protocol was thoroughly explained to the patient in the language of their choice (English, Twi, or Ga). If the patient was interested, they provided written informed consent to participate. The research team reviewed hospital records, including the ECV and delivery record books, on a weekly basis to ensure no patients were missed in the recruitment process.

After enrollment, the research team directly observed each patient’s antenatal care visits to collect data on the counseling related to their malpresentation diagnosis and ECV procedure if performed. Delivery information was collected postpartum via the patient’s pregnancy care booklet. Malpresentation counseling was classified as “performed” if the options of ECV and CD were discussed with the patient; vaginal breech delivery discussions were not included. The counseling method and information given was performed by the patients’ clinician per their standard clinical practices; the research team did not intervene. Patients were able to unenroll at any time and the research team did not participate in the patients’ care.

In the postpartum period, all enrolled patients were requested to participate in a semi-structured interview using an interview guide developed by the authors based on the Theoretical Framework of Acceptability to understand their perspectives and decision-making around accepting, declining, or not being offered ECV (see Additional files 1 and 2).^5^ The interviews were conducted privately in person in the language of their choice (English, Twi, or Ga). Enrolled patients’ clinicians, including trainees, were also requested to participate in a semi-structured interview using an interview guide developed by the authors based on the Theoretical Framework of Acceptability to understand their perspectives and decision-making around offering or not offering ECV (see Additional file 3).^5^ Clinicians were only invited to interview once, regarding their first patient enrolled in the study; information regarding any additional patients who were enrolled was not addressed. Interviews were conducted privately in person in English. All interviews were audio-recorded. Recordings were translated and back-translated from Twi or Ga to English (if necessary) to ensure consistency across languages and transcribed verbatim. Recordings in English were transcribed verbatim with GoTranscript (Lewes, DE, USA). The final numbers of interviews for both patients and clinicians were determined by thematic saturation of the data and/or completion of the recruitment process.

Quantitative data was collected in Research Electronic Data Capture (REDCap) and analyzed with Stata v18 (College Station, TX, USA). For data analysis, age (in years) was re-coded as 18–34 and 35 or greater; relationship status was re-coded as relationship or single; highest completed education was re-oded as junior high school or less, senior high school, or tertiary; partner highest completed education was re-coded as senior high school or less or tertiary given low sample size; and the differences between the ECV-performing clinician and the counseling clinician were manually compared and coded as yes or no. No patient’s medical history was complicated by gestational diabetes in a previous pregnancy, seizure disorder, respiratory diseases other than asthma, mental illness, heart disease/failure, tuberculosis, or smoking; therefore, these conditions were not included in the analysis. We performed descriptive and inferential statistics including cross-tabulation with Chi-square analysis or Fisher’s exact test for categorical variables and independent samples t-test or analysis of variance for continuous variables. Statistical significance was defined at p < 0.05.

We imported the patients’ qualitative data into Dedoose 10.0.35 (Manhattan Beach, CA, USA) for coding. Two research team members independently reviewed the transcripts to develop parent and child codes and iteratively developed a finalized codebook, which was reviewed by the remaining research team members for completion. Two research team members independently coded each transcript in Dedoose using the finalized codebook. Coding discrepancies were discussed and resolved on a weekly basis to ensure consistency throughout the coding process. We conducted thematic analysis using the thematic networks technique to understand patient decision-making around malpresentation and ECV.^6^ This process was replicated for the clinicians’ qualitative data.

## Results

During the study period, 57 patients were identified at 36 weeks’ gestation as eligible for ECV. Six (10.5%) patients declined recruitment. Of the 51 (89.5%) patients who enrolled, five (9.8%) were lost to follow-up: three were no longer interested in being in the study, one patent was no longer interested in pursuing care at KBTH, and one provided an incorrect phone number for follow-up. A total of 46 (80.7%) eligible patients ultimately enrolled and were followed to delivery (Table 1). All patients had national health insurance. Three patients, who had a history of one previous CD, had this first CD due to malpresentation.

**Figure 1:**
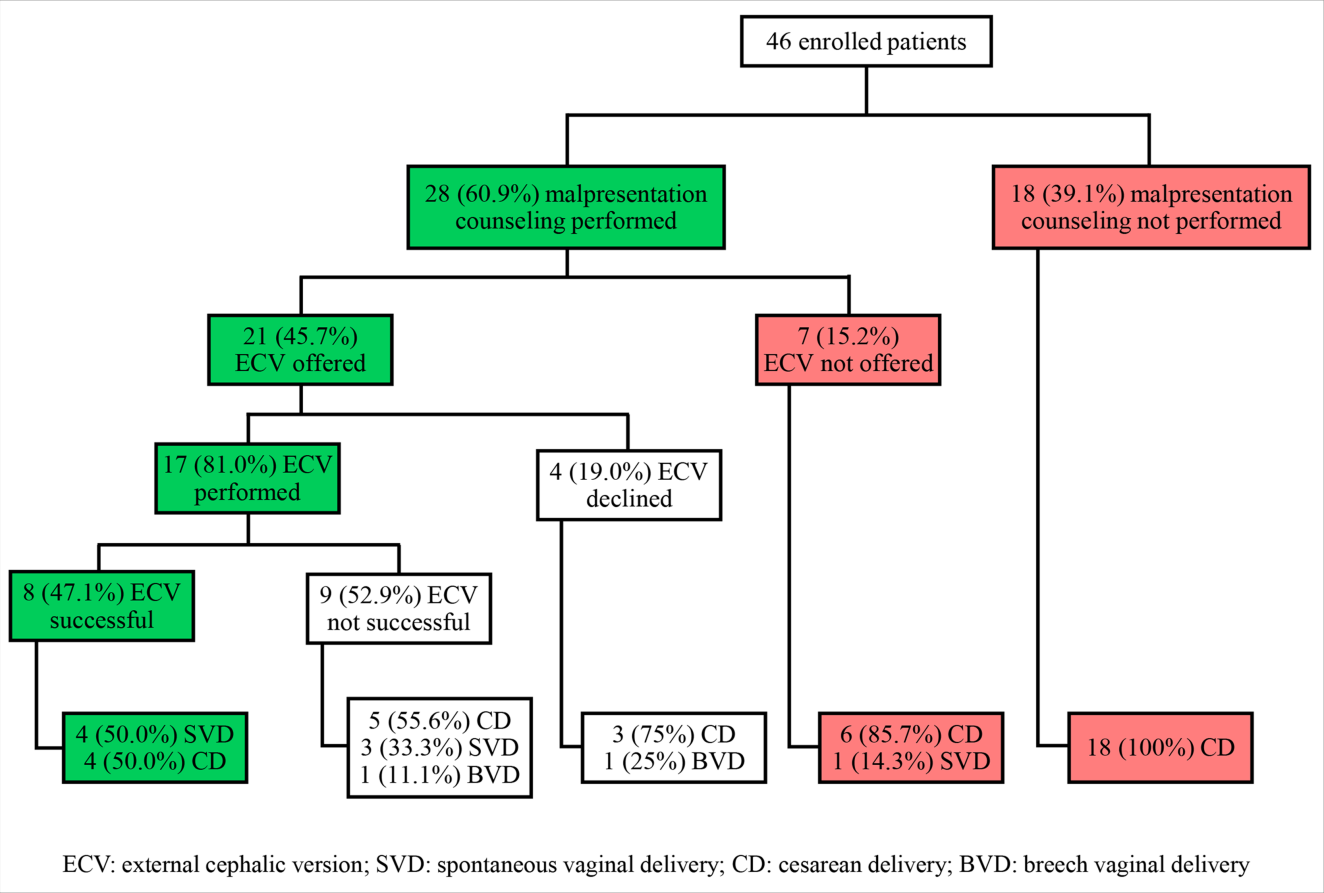
Enrolled participant outcomes

Out of the 46 enrolled patients, ECV was discussed with 28 (60.9%) and offered to 21 (45.7%). Four (19.0%) patients who were offered ECV declined. Eight (47.1%) of the 17 ECVs performed were successful and none reverted to malpresentation before delivery (Fig. 1).

**Figure 2:**
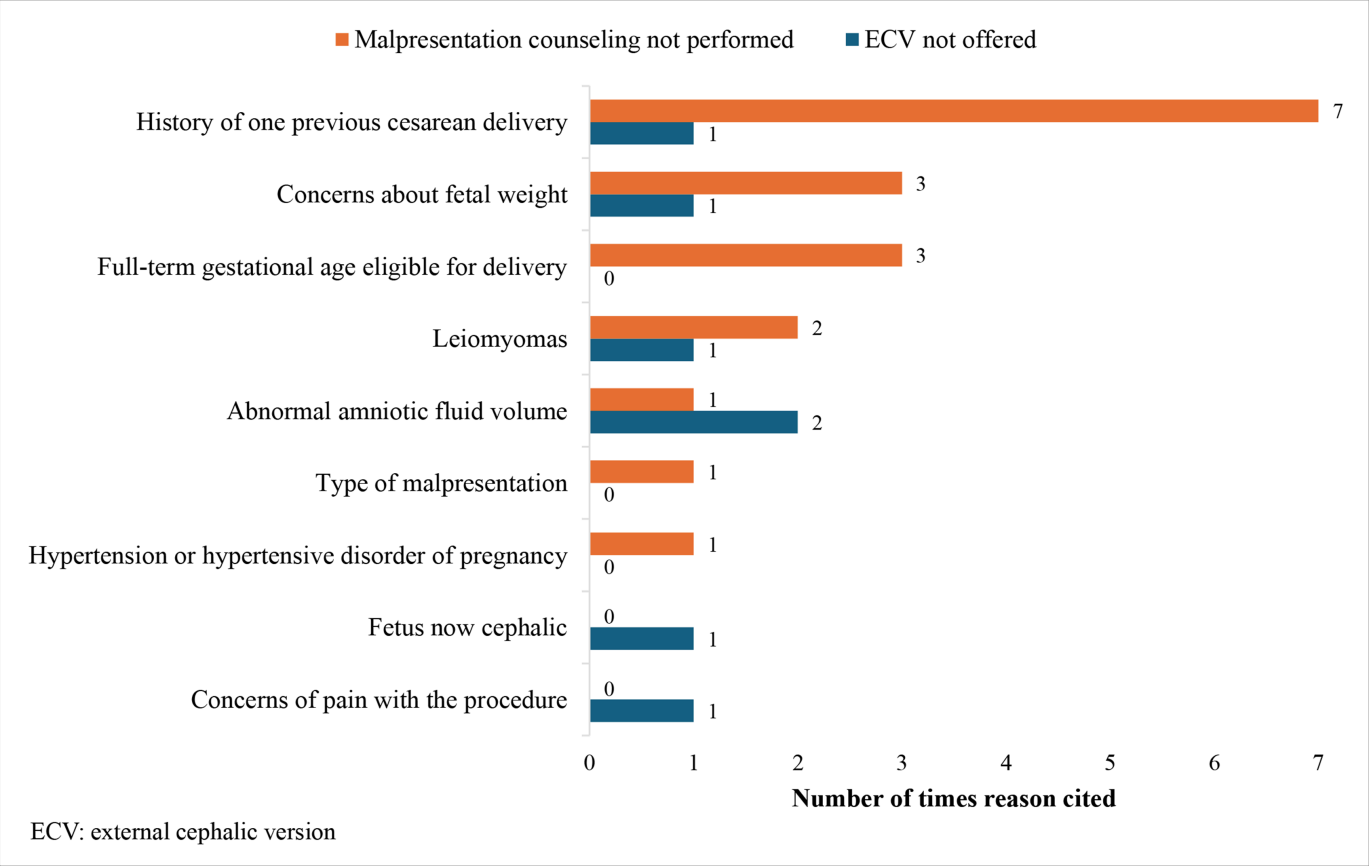
Reasons stated by clinicians for recommending cesarean delivery

Figure 2 demonstrates the various reasons clinicians stated for recommending CD over ECV during the counseling session, as observed by the research team. Upon individual review of the ultrasound findings by the research team, no fetuses were identified as growth restricted or having macrosomia to the extent CD was required. Additionally, there were no cases where fibroids were considered a contraindication to a trial of vaginal delivery.

When comparing reasons for malpresentation counseling not being performed, history of one previous CD was a significant factor (Table 2). Of the 46 enrolled patients, eight (17.4%) had a history of one previous CD; however, only one (12.5%) received malpresentation counseling. Malpresentation-related history and pregnancy complications related to current or previous fetal anatomical abnormality, diabetes mellitus or gestational diabetes, sickle cell disease, HIV, asthma, Rh negative, postpartum hemorrhage in previous pregnancy, and fetal or neonatal demise in previous pregnancy were too small for comparisons.

Of the 46 enrolled patients, 35 (76.1%) completed an interview regarding their malpresentation and ECV counseling, ECV procedure if performed, and postpartum experience; of these, 16 (45.7%) were never offered ECV and proceeded to deliver by CD. The other 19 patients (54.3%) were offered ECV: 15 (78.9%) elected to pursue ECV, resulting in eight (53.3%) spontaneous vaginal deliveries and seven (46.7%) CDs; and four (21.1%) decided against pursuing ECV, resulting in one (25.0%) vaginal breech delivery and three (75.0%) CDs. Twenty of these patients’ clinicians completed an interview regarding their malpresentation and ECV counseling and ECV procedure if performed: 10 (50.0%) offered ECV and 10 (50.0%) did not offer ECV to these patients (Table 3).

Regarding decision-making around whether to offer and/or pursue ECV, patients refrained from pursuing ECV if they had gaps in understanding the procedure and its complications but were more likely to pursue it when supported by partners, friends, and clinicians. Although, clinicians demonstrated appropriate knowledge to counsel patients regarding the procedure and its complications. Both patients and clinicians were motivated by their desire to avoid the financial cost, long recovery time, and future complications of undergoing a CD. Overall, patients and clinicians had a positive perception of ECV and would endorse it regardless of whether the procedure resulted in a vaginal delivery or not (Table 4).

Despite clinicians valuing ECV, they highlighted barriers preventing them from offering ECV to more patients, including patients with a history of one prior CD, hospital resource barriers to performing ECV, and limited training opportunities to learn ECV procedural skills (Table 5).

Clinicians and patients were comfortable with midwives performing ECV if they were appropriately trained to do so.

Overall, medical counseling, trust in the health care system and professional, and personal values were fundamental motivators to patients as they began to make their decision on whether to have ECV or not.

**Figure 3:**
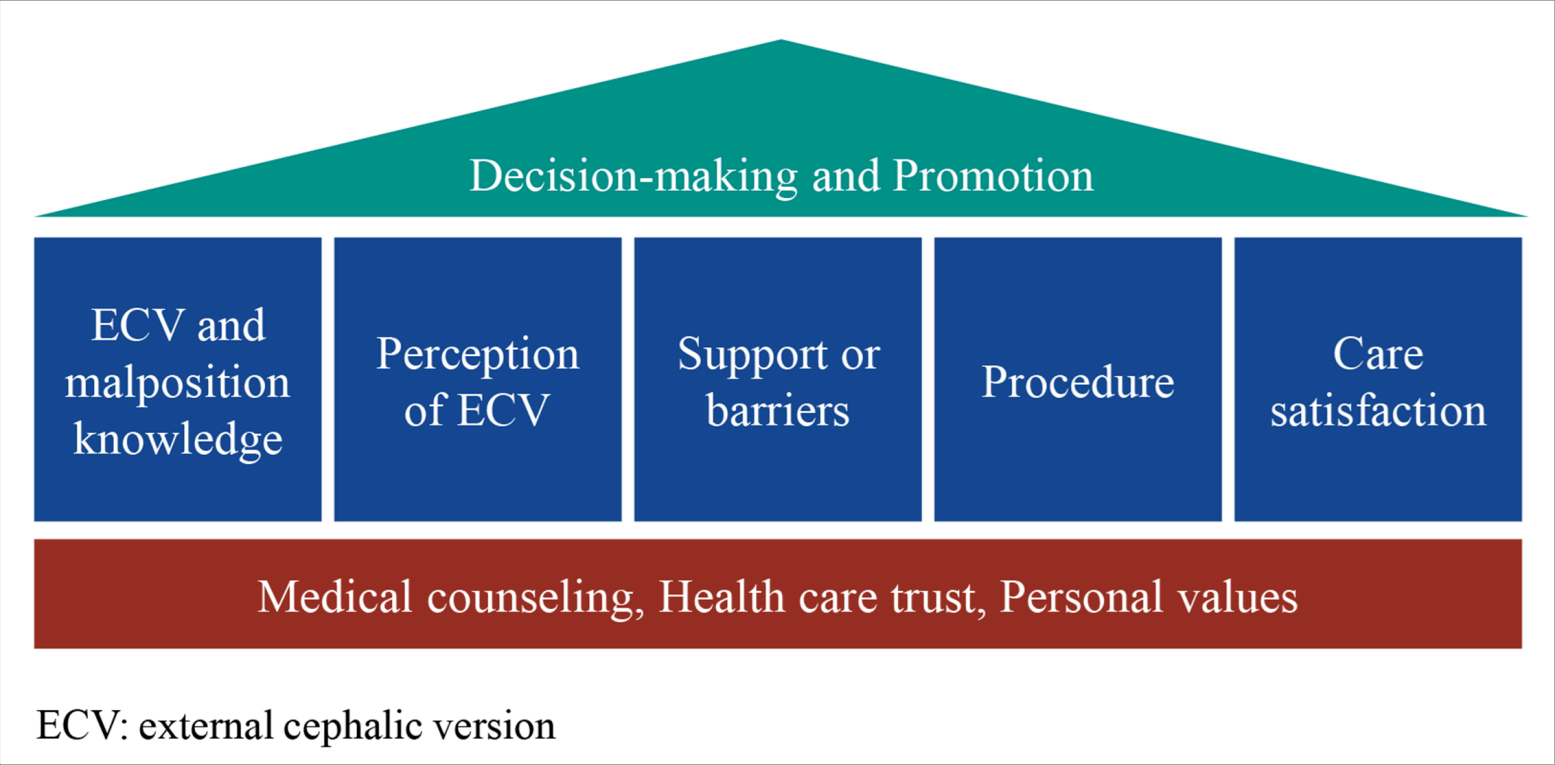
Summary of how patients navigate the decision process for ECV

Knowledge of ECV and malpresentation, perception of ECV, support or barriers experienced, experience of the ECV procedure, and care satisfaction played influential roles in a patient’s final decision to pursue and promote ECV to others (Fig. 3).

## Discussion

Among 46 enrolled patients who were eligible for ECV, only 21 (45.7%) patients were offered the procedure. Though a patient having a history of one previous CD was a significant factor in clinicians not offering ECV, evidence demonstrates that this does not increase the risk of ECV complications, nor does it decrease the success rate of an ECV. It is generally recommended to offer ECV to patients who are otherwise eligible for trial of labor after cesarean.^2,3,8,9^ Though other concerns cited by clinicians such as fetal weight, gestational age, leiomyomas, abnormal amniotic fluid volume, and type of malpresentation may factor into the success of ECV, there is conflicting data regarding the predictive value of these factors; therefore, decisions to not offer ECV should not be based solely on these factors.^2,3,10–17^ While caution must be used with hypertensive patients to ensure stability can be achieved while awaiting a vaginal delivery, hypertension itself has not been noted as a contraindication to ECV.^2,3^ Additionally, although ECV can be a painful procedure—information that should be included in patient counseling—most patients can tolerate this procedure and can be offered ECV.^2,18^ Finally, while limited resources in the hospital for fetal monitoring was cited as a factor for not offering ECV, and although the non-stress test is the gold standard for monitoring, alternatives such as ultrasound may be used to continue to offer this overall low-resource procedure.^2,3^

The biggest contributing factor identified by patients for being concerned and/or not pursuing ECV was a gap in understanding the procedure and its potential complications. This is aligned with other studies conducted in Australia, England, and the United States.^19–21^ Similar to our finding that patients are more likely to make their decision about pursuing ECV with family, friends, and clinicians, studies among patients in Nigeria, England, and Australia have demonstrated the same.^19,20,22^ Conversely, another study in Austria demonstrated that family and friends were a reason patients decided against pursuing ECV.^23^ Interestingly, in our study, some patients felt comfortable

and motivated to make their own decision without partner support and/or input, which does not align with other studies performed in Ghana which highlighted the strong partners role in health care decision-making.^24,25^ In studies completed in the United States and England, knowledge of the success rate of the ECV procedure played a large role in patient decision-making, which we did not find in our study.^20,21^

In our study, patients and clinicians valued ECV as a way to prevent CD, which aligns with patients and clinicians in England, the United States, and other studies among Ghanaians.^20,21,26^ While patients in our study desired to promote ECV to others even if it was not successful and resulted in a CD, this is unique compared to studies of patients in Austria and Australia.^19,23^

There are opportunities to improve clinician knowledge on evidence-based indications for offering ECV to encourage more patients who can benet from this procedure, as well as to implement training on counseling practices to discuss the risks, benefits, and alternatives to ECV that resonate with patients and aid in their decision-making. There may be a bene t to encourage the involvement of patients’ families and other support persons throughout the counseling and decision-making process. Revising hospital guidelines for evidence-supported fetal monitoring during and after the procedure may reduce the burden on hospital resources that currently limit ECV from being offered. Additionally, these findings demonstrate an opportunity to train clinicians, such as in a simulation-based curriculum, to improve the knowledge, confidence, and sustainability of ECV practices.^27^ Importantly, ECV training for midwives may be beneficial to ensure patients have greater access to ECV services.

### Strengths and limitations

To our knowledge this is the first study to explore and evaluate ECV practices in Ghana. However, this study was limited to KBTH, which as the largest teaching hospital is more likely to be performing evidence-based practices than other hospitals. Additionally, the Department of Obstetrics and Gynecology was informed of this study and research assistants were present at every counseling visit or ECV procedure, which may have biased counseling patterns and/or interview responses for patients whose pregnancies were complicated by malpresentation given that it was known we were evaluating malpresentation practices. Patients who declined to participate or were lost to follow-up may have had other experiences.

## Conclusions

With increasing CD rates in sub-Saharan Africa, preventing CDs is paramount to reducing maternal and neonatal morbidity and mortality, aligned with the United Nations’ Sustainable Development Goal three.^4,28,29^ ECV is a proven, low-risk, low-resource, and cost-effective procedure, when performed by a skilled clinician, to reduce CD rates for malpresented fetuses.^2,30–32^ This study demonstrates that there is an opportunity to improve counseling and utilize evidence-based indications to offer ECV to patients whose pregnancies are complicated by fetal malpresentation. There is also an important opportunity to ensure clinicians, and potentially midwives, are trained to perform ECV. Addressing these opportunities successfully could improve the numbers of health care professionals who practice safe and effective ECV in sub-Saharan Africa and potentially decrease rates of avoidable CD and their associated morbidity and mortality.^2,3^

## Supplementary Material

This is a list of supplementary fi les associated with this preprint. Click to download.

• Additionalfile1.docx

• Additionalfile2.docx

• Additionalfile3.docx

## Figures and Tables

**Table 1:** Demographics of participants who completed the study (N = 46)

Characteristic	n (%) or mean ± SD	
Patient age (years)		
18–34		28 (60.9)
35 or older		18 (39.1)
Relationship status		
Married or in relationship		32 (69.5)
Single		14 (30.5)
Highest completed education		
Junior high school or less		15 (32.6)
Senior high school		18 (39.1)
Tertiary		13 (28.3)
Malpresentation-related history		
Personal history of malpresentation		7 (15.2)
Personal history of ECV		1 (2.2)
Family history of malpresentation		3 (6.5)
Family history of ECV		2 (4.4)
Pregnancy complications		
History of one previous cesarean delivery	8 (17.4)	
Leiomyomas	3 (6.5)	
Hypertension or hypertension disorder of pregnancy	4 (8.7)	
Current or previous fetal anatomical abnormality	3 (6.5)	
Recurrent pregnancy loss	9 (19.6)	
Diabetes mellitus or gestational diabetes	1 (2.2)	
Sickle cell disease	1 (2.2)	
HIV	3 (6.5)	
Asthma	1 (2.2)	
Rh factor negative	1 (2.2)	
Postpartum hemorrhage in previous pregnancy	3 (6.5)	
Fetal or neonatal demise in previous pregnancy	4 (8.7)	
Gravidity	3 ± 1	
Parity	1 ±1	
Body mass index (kg/m^2^)	28.1 ±5.2	

ECV: external cephalic version

**Table 2:** Comparison of factors in relation to malpresentation counseling performed and ECV offered

Factor	Malpresentation Counseling Performed			ECV Offered		
	Yes (n=28)	No (n = 18)	p-value	Yes (n = 21)	No (n=7)	p-value
Patient age (years)			0.518			0.662
18–34	16 (57.1)	12 (66.7)		11 (52.4)	5 (71.4)	
35 or older	12 (42.9)	6 (33.3)		10 (47.6)	2 (28.6)	
Relationship status			0.732			N/A^a^
Married or in relationship	20 (71.4)	12 (66.7)		15 (71.4)	5 (71.4)	
Single	8 (28.6)	6 (33.3)		6 (28.6)	2 (28.6)	
Highest completed education			0.339			N/A^a^
Junior high school or less	9 (31.1)	6 (33.3)		6 (28.6)	3 (42.8)	
Senior high school	13 (46.4)	5 (27.8)		11 (52.4)	2 (28.6)	
Tertiary	6 (21.5)	7 (38.9)		4 (19.0)	2 (28.6)	
Partner age^b^ (years)			0.696			N/A^a^
18–34	5 (25.0)	4 (33.3)		4 (26.7)	1 (20.0)	
35 or older	15 (75.0)	8 (66.7)		11 (73.3)	4 (80.0)	
Partner highest completed education^2^			0.141			N/A^a^
Senior high school or less	12 (60.0)	3 (25.0)		9 (60.0)	3 (60.0)	
Tertiary	8 (40.0)	9 (75.0)		6 (40.0)	2 (40.0)	
Pregnancy complications						
History of one previous CD	1 (3.6)	7 (38.9)	0.004	0 (0.0)	1 (14.3)	N/A^a^
Leiomyomas	1 (3.6)	2 (11.1)	N/A^a^	0 (0.0)	1 (14.3)	N/A^a^
Hypertension or hypertensive disorder of	1 (3.6)	3 (16.7)	N/A^a^	1 (4.8)	0 (0.0)	N/A^a^
pregnancy						
Recurrent pregnancy loss	5 (17.9)	4 (22.2)	0.721	5 (23.8)	0 (0.0)	0.290
Gravidity	3 ± 1	3 ± 1	0.865	3 ± 1	2 ± 1	0.461
Parity	1 ± 1	1 ± 1	0.902	1 ± 1	1 ± 1	0.646
Body Mass Index (kg/m^2^)	28.2 ± 5.4	27.9 ± 5.1	0.857	28.3 ± 5.6	27.9 ± 5.4	0.867
Ultrasound findings informing counseling						
Gestational age (weeks)	37.1 ± 1.7	36.9 ± 1.6	0.692	36.9 ± 1.7	37.8 ± 1.9	0.201
Presentation			N/A^a^			N/A^a^
Breech	24 (85.7)	15 (83.3)		18 (85.8)	6 (85.7)	
Transverse	4 (14.3)	3 (16.7)		3 (14.2)	1 (14.3)	
Placenta location			0.507			N/A^a^
Anterior	11 (39.3)	9 (50.0)		9 (42.9)	2 (28.6)	
Fundal	6 (21.4)	5 (27.8)		3 (14.2)	3 (42.8)	
Posterior or fundal	11 (39.3)	4 (22.2)		9 (42.9)	2 (28.6)	
Amniotic fluid volume			N/A^a^			N/A^a^
Normal	25 (89.3)	16 (88.9)		20 (95.2)	5 (71.4)	
Abnormal	3 (10.7)	2 (11.1)		1 (4.8)	2 (28.6)	
Estimated fetal weight (grams)	2963.036 ± 484.082	3010.500 ± 546.694	0.759	2901.810 ± 402.565	3146.714 ± 673.154	0.252
Counseling specifics						
Gestational age (weeks)	37.5 ± 1.2	37.3 ± 1.5	0.618	37.4 ± 1.1	37.8 ± 1.5	0.478
Level of primary counseling clinician			0.570			N/A^a^
House officer	2 (7.1)	0 (0.0)		1 (4.8)	1 (14.3)	
Junior resident	5 (17.9)	4 (22.2)		5 (23.8)	0 (0.0)	
Senior resident	14 (50.0)	7 (38.9)		10 (47.6)	4 (57.1)	
Consultant	7 (25.0)	7 (38.9)		5 (23.8)	2 (28.6)	

Data presented as n (%) or mean ± SD

a count too small for analysis

b n does not include entirety, as category is not applicable to all

ECV: external cephalic version; CD: cesarean delivery

**Table 3:** Demographics of clinicians who were interviewed (n = 20)

Level of Primary Counseling Clinician	ECV Offered (n = 10)	ECV Not Offered (n = 10)
House Officer	1 (10)	0 (0)
Junior Resident	3 (30)	2 (20)
Senior Resident/Fellow	4 (40)	6 (60)
Consultant/Attending	2 (20)	2 (20)
Data presented as n (%)		

ECV: external cephalic version; CD: cesarean delivery

**Table 4:** Factors influencing patient and clinician decision-making

Theme	Representative Patient Quotes	Representative Clinician Quotes
Factors against pursing ECV		
**Gaps in ECV understanding between clinicians and patients after counseling**	"I didn’t remember [the doctor explaining any disadvantage or advantage of ECV] because I was already a bit anxious about the procedure." (ECV accepted, CD)	"As I said, I have to make sure that there is no contraindication. Once the baby is the right size, everything else is normal, so I will tell the patient that she's a good candidate for the procedure, and then what the procedure involves, how we're going to turn the baby for the head into the pelvis, and then what it involves. How we might use tocolytic or we might not use, whether we are going to use tocolytics or not. We usually will do a CTG before and after. We will tell the patient. We also let the patient know that after the procedure, should there be any abnormality, then we might proceed to deliver." (ECV offered, senior resident)
	"[The clinician's said ECV is] the turning of the baby. But is that even possible?" (ECV declined, vaginal breech delivery)	
**Gaps in understanding of ECV complications between clinicians and patients after counseling**	"I donť recount him explaining the complications about the procedure to me, however, he told me if the procedure was successful, I will be admitted and be able to deliver vaginally." (ECV accepted, SVD)	"The common complications, the fetal bradycardia. After that we also put them on the continuous fetal monitoring with CTG to monitor the fetus heart, whether there is any bradycardia or any fetal distress. You can also have a cord entanglement or a true knot. The cord can knot impeding blood supply from the mother to the fetus. You can also have placenta abruption, or the patient can also rupture its membrane as well. These are some of the complications... you can have a uterine rupture as well. These are some of the complications associated with it. We tell our patients all these." (ECV offered, senior resident)
	"All [the doctors] were talking about [complications in] their terms but I didnť really understand." (ECV accepted, CD)	
**Factors for pursing ECV**		
**Support from family, friends, and further clinician counseling**	"My cousin also had an ECV which was successful, so I decided also to do it. Her testimony about ECV also inspired my decision even though I was a bit hesitant at first." (ECV accepted, CD)	"…someone else has told them that iťs going to fail and iťs painful, or those things. Those are some of the challenges. You have to try to dispel those myths and then counsel them appropriately." (ECV not offered, junior resident)
	Some people encouraged me, others did not. Some said they have never heard about malpresentation; hence it could be that I have offended someone. Others encouraged me saying, I am not the first person to go through this. On the labor ward, before the ECV, I asked one of the doctors if I was the first person to have this procedure. The doctor said no and further mentioned that those on the ward with me were also waiting to have ECV done. That really encouraged me." (ECV accepted, SVD)	
**Spouses participated in conversations on malpresentation management**	"I wouldnť [have agreed with my husband if he told me not to take part in the ECV] at all, because he doesnť know what in going through." (ECV not offered, CD)	"Sometimes [the patients] say they want to go home, discuss with their husbands and come back." (ECV not offered, consultant)
	"When I got home, I explained everything to my husband, and he said ok if they can do it then I should go ahead with it…If he didnť agree I wouldnť have gone ahead with it." (ECV accepted, CD)	
**Pursing ECV to avoid CD**	"Well, everyone has a choice. Some people do not have any complications but prefer C/S. I prefer the ECV because after delivery, you do not have to deal with any pain, unlike C/S. With ECV, the pain is felt on the day it is done, and it is little. Once baby is delivered you are fine." (ECV accepted, SVD)	"I think if someone has a successful ECV, it saves them having to go through a CS with all these future complications, and then also having to go through an assisted vaginal delivery. I would rather want to avoid that, so if you get an ECV done, yes, thaťs good. I think it helps them, or it impacts them positively." (ECV not offered, senior resident)
	"Do your normal delivery. After some weeks, you'll be fine, you'll do your things but when you do the section, you canť do so many things for some months, there are some things you canť even do." (ECV accepted, SVD)	"It feels good to be able to offer an ECV to a patient just so that I can reduce the primary cesarean section rate, reduce the economic burden on the patient, and then also make them have shorter stay in the hospital when they have a vaginal delivery." (ECV not offered, senior resident)
**Positive perception and endorsement of ECV**	"I will definitely recommend [ECV] to anyone by sharing the testimony of my experience." (ECV accepted, SVD)	"Most often than not the women are very, very satisfied. They're able to deliver naturally." (ECV offered, senior resident)
	"…my opinion is, just do the ECV, just do it, do your normal delivery. Because me, this, I call it a miracle." (ECV accepted, SVD)	"The ECV offers them an opportunity to do that safely and successfully. A lot of patients, once they've been counseled and they accept it, they are happy, especially when iťs successful, because they are motivated, our patients here are motivated to deliver vaginally. They are usually happy about it." (ECV offered, senior resident)

ECV: external cephalic version; CD: cesarean delivery; SVD: spontaneous vaginal delivery

**Table 5:** Clinician barriers to offering ECV

**Theme**	**Representative Clinician Quotes**
**Patients with a history of one prior CD**	“When they come, when the presentation is breech, we counsel them on the condition and we give them three options. It depends also on the patient. If it’s a primi, someone who has never delivered before, a straight Cesarean section. If the patient has had vaginal delivery before, they have three options. Either we do the ECV for them, or we do Cesarean section, or we do assisted breech delivery. With the breech delivery, when they come, imagine the patient that come on 35 weeks and is breech, we’ll counsel her on her option.” (ECV offered, senior resident)
	“… Here in Korle-Bu, we do not try ECVs for people who have had previous CS because the first thing is, you don’t even know where the person did the CS. You don’t know who did the CS. I’m not sure if it was a lower uterine cut, or the cut is high up in the uterine bone. You cannot verify that the scar is really intact for you to try an ECV. It’s a high risk of rupture.” (ECV offered, junior resident)
**Hospital resource limitations**	“When there is no bed in the labor ward, it means you can’t do the ECV. The ECV has to be postponed.” (ECV not offered, senior resident)
	“At times, if you’re not having enough CTGs or fetal monitors around, that’s when the challenge comes in because it needs to monitor the fetal heart rate for some time.” (ECV offered, junior resident)
**Limited training opportunities despite an interest in training**	“I think it needs to be more one-on-one, because of how large Korle-Bu is. When I was in medical school, we were like 20 in a room observing one ECV. Same thing, even being a house officer, we still have students around. I feel there’s not much- you can’t really learn much. The one-on-one would be better, or at least even three on one or two on one. More targeted, specific, isolated training would be helpful, I think.” (ECV offered, house officer)
	“I haven’t had a training. I’ve only observed and have read about the training” (ECV not offered, junior resident)
**Other Ghanaian hospitals do not perform ECV**	“I was coming from a place that we were not offering ECV at all to our patients. When you come with a normal lie, when you come with a breech, a transverse lie breech, the only option available to us was to do cesarean section.” (ECV offered, junior resident)
	“Other facilities, some may just go straight and do a cesarean section because they don’t have the skill to perform the ECV. When you come and there is a malpresentation, either a transverse lie or a breech presentation, then they opt for cesarean section as the only mode of delivery of their baby. That is the difference from some other facilities as compared to Korle-Bu where residents are trained to perform the ECV.” (ECV offered, senior resident)

ECV: external cephalic version; CS: cesarean section

## Data Availability

The data that support the findings of this study are available from the corresponding author, SAO, upon reasonable request.

## Abbreviations

CD: cesarean delivery
LMIC: low- and middle-income countries
KBTH: Korle Bu Teaching Hospital
ECV: external cephalic version
